# Genetic and environmental influences on conduct and antisocial personality problems in childhood, adolescence, and adulthood

**DOI:** 10.1007/s00787-017-1014-y

**Published:** 2017-06-21

**Authors:** Laura W. Wesseldijk, Meike Bartels, Jacqueline M. Vink, Catharina E. M. van Beijsterveldt, Lannie Ligthart, Dorret I. Boomsma, Christel M. Middeldorp

**Affiliations:** 1 Department of Biological Psychology, VU University Amsterdam, Van der Boechorststraat 1, 1081 BT Amsterdam, The Netherlands; 2 EMGO+ Institute for Health and Care Research, Amsterdam, The Netherlands; 3 Neuroscience Campus Amsterdam, Amsterdam, The Netherlands; 4 Behavioural Science Institute, Radboud University, Nijmegen, The Netherlands; 5 Department of Child and Adolescent Psychiatry, GGZ inGeest/VU University Medical Center, Amsterdam, The Netherlands

**Keywords:** Conduct problems, Antisocial personality problems, Longitudinal, Heritability, Shared environment, Twins

## Abstract

Conduct problems in children and adolescents can predict antisocial personality disorder and related problems, such as crime and conviction. We sought an explanation for such predictions by performing a genetic longitudinal analysis. We estimated the effects of genetic, shared environmental, and unique environmental factors on variation in conduct problems measured at childhood and adolescence and antisocial personality problems measured at adulthood and on the covariation across ages. We also tested whether these estimates differed by sex. Longitudinal data were collected in the Netherlands Twin Register over a period of 27 years. Age appropriate and comparable measures of conduct and antisocial personality problems, assessed with the Achenbach System of Empirically Based Assessment, were available for 9783 9–10-year-old, 6839 13–18-year-old, and 7909 19–65-year-old twin pairs, respectively; 5114 twins have two or more assessments. At all ages, men scored higher than women. There were no sex differences in the estimates of the genetic and environmental influences. During childhood, genetic and environmental factors *shared* by children in families explained 43 and 44% of the variance of conduct problems, with the remaining variance due to unique environment. During adolescence and adulthood, genetic and *unique* environmental factors equally explained the variation. Longitudinal correlations across age varied between 0.20 and 0.38 and were mainly due to stable genetic factors. We conclude that shared environment is mainly of importance during childhood, while genetic factors contribute to variation in conduct and antisocial personality problems at all ages, and also underlie its stability over age.

**Electronic supplementary material:**

The online version of this article (doi:10.1007/s00787-017-1014-y) contains supplementary material, which is available to authorized users.

## Introduction

Conduct problems in children and adolescents and antisocial personality problems in adults involve a variety of repetitive and persistent behaviors that violate the rights of others or societal norms or rules, such as aggression to people, destruction of property, theft, or violations of rules [1]. Conduct problems during childhood may be the developmental precursor for adult antisocial personality problems and are significantly associated with adverse adult outcomes related to health, crime and conviction, and financial and personal functioning [2–6]. Worldwide, childhood, and adolescent conduct problems and adult antisocial personality problems pose a challenge to societies and health care, with prevalence rates ranging between 1 and 4% in the general population [7–9]. It is important to get insight into risk factors for conduct and antisocial personality problems, especially into the factors influencing the stability over age. We, therefore, present a longitudinal twin study (*N* = 17,513 twin pairs) following twins from age 8 until adulthood (30 years on average).

Twin studies allow the estimation of influences of genetic, shared, and unique environmental factors on individual differences in behavior and on stability over ages. Most twin studies performed cross-sectional analyses on conduct and antisocial personality problems. Meta-analyses of these studies have estimated the proportion of the variation explained by genetic factors to be between 32 and 60% for children, between 45 and 50% for adolescents, and one meta-analysis provided a heritability estimate of 49% for adults [10–13]. The contribution of the shared environment, also referred to as the ‘common’ familial environment, has been estimated between 10 and 20% in childhood, between 10 and 17% in adolescence, and 14% in adulthood [10–13]. In twin studies, the remaining variation, that is not due to genetics or the shared environment, is attributed to unique environmental influences, which also includes measurement error. Thus, all meta-analyses agree upon the importance of genetic factors, and also agree upon a lower estimate for the contribution of shared environment.

Longitudinal twin studies on conduct and antisocial personality problems reported stability over ages to be mainly due to genetic factors [14–17], and three studies reported an additional small influence of the shared environment on the stability across ages [15–17]. However, research on antisocial personality problems in later adulthood, i.e., above 24 years, is underrepresented. Furthermore, the sex differences in prevalences for conduct and antisocial personality problems [16, 18] lead to the questions whether the influence of genetic and environmental influences also is different for males and females (i.e., quantitative sex differences) and whether there the same or different genes play a role (i.e., qualitative sex differences). Some twin studies have reported qualitative sex differences for conduct and antisocial problems [16, 19], while others have not [15, 17, 20–23], just as some studies have detected quantitative sex differences [15, 17, 19, 21, 24], and others have not [12, 16, 20, 22, 25]. As the effect of sex might differ across age, we address this question by analyzing large samples of twins ranging from 8 to 65 years.

The aim of the present study was to elucidate the genetic architecture of conduct and antisocial personality problems by analyzing a large longitudinal data set with observations of 9–10-year-old twins (childhood), 13–18-year-old twins (adolescence), and 19–65-year-old twins (adulthood). The data, collected over a 27 year period in the Netherlands Twin Register, offer the opportunity to examine the following questions: (1) what is the heritability of conduct problems in childhood and adolescence and of antisocial personality problems in adulthood? (2) What is the longitudinal stability across age and which factors contribute to the stability? (3) Do genetic and environmental factors interact with sex? We analyzed the DSM-oriented conduct problems scales of the Child Behavior Checklist (CBCL) and the Youth Self-Report (YSR) and the antisocial personality problem scale of the Adult Self-Report (ASR) belonging to the Achenbach System of Empirically Based Assessment (ASEBA) that consist of similar sets of items across ages [26, 27].

## Methods

### Subjects

The Netherlands Twin Register (NTR) includes a register for young twins, the YNTR [28], and for adult twins, the ANTR [29]. Since 1986, parents can register their young twins shortly after birth with the YNTR and will then receive a survey when their twins are around 1, 2, 3, 5, 7, 10, and 12 years. Between 2005 and 2013, twins themselves were asked to complete a survey when they reached ages 14 and 16 years. At the age of 18 years, twins enroll into the ANTR. The ANTR started in 1991 by recruiting adolescent twins and their family members through city councils and subsequently added adults through volunteer registration [29]. The NTR data collection is prospective, with data collected when twins reach a particular age. Therefore, more data are available from twin pairs at younger ages in the YNTR. Due to financial constraints, there was no data collection of the survey for 10 years in 2008 [28]. Until 2008, the survey for 10 years was mailed to both parents of twins around the 10th birthday of the twins. From 2009, the survey was mailed around the 9th birthday of the twins. For the current study, maternal ratings of 9–10-year-old young twins from birth cohorts 1986–2004 were included. Average age was 10 years. The sample included 60 pairs between age 8.7–9 years and 10 pairs who had reached age 12 years when their parents completed the survey, throughout the paper, we refer to this group as 9–10-year-olds. Paternal reports were not included; an earlier study of these twins at age 7 showed that heritability estimates for conduct problems did not differ between paternal and maternal reports [30]. For the 13–18-year-old adolescent twins, self-report data from birth cohorts 1986–1999 were analyzed. Self-report data from 19- to 65-year-old adult twins were collected in the ANTR in 1997, 2000, 2009–2012, or 2013–2014. If adolescent twins had completed multiple surveys, the survey completed by both twins closest to age 16 was selected. For adult twins, a preference was given to the survey that was completed by both twins, closest to age 40. The final sample contained 17,513 twin pairs, including 9783 child twin pairs (9702 complete and 81 incomplete twin pairs on average 10 years), 6839 adolescent twin pairs (5107 complete and 1732 incomplete twin pairs, on average 15.77 years), and 7909 adult twin pairs (4752 complete and 3157 incomplete twin pairs on average 29.39 years). Table 2 presents sample sizes per zygosity-by-sex group. There were 3283 complete and 694 incomplete twin pairs with data in childhood and adolescence. Between adolescence and adulthood, the overlap was 1135 complete and 1163 incomplete twin pairs. For 1412 complete and 1253 incomplete twin pairs, the NTR had data in childhood and adulthood. Overall, there were 985 complete and 1913 incomplete twin pairs with data available in childhood, adolescence, and adulthood.

### Phenotypes

Conduct and antisocial personality problems were measured with the age appropriate versions of the questionnaires belonging to the Achenbach System of Empirically Based Assessment (ASEBA), i.e., the Child Behavior Checklist (CBCL), the Youth Self-Report (YSR), and the Adult Self-Report (ASR). In all three instruments, items are rated on a three-point scale (0–2; not true, somewhat true, very true). The conduct problem scales of the CBCL and YSR are based on a similar set of items that only differ in the phrasing depending on whether the parent is asked to rate his or her child (“Gets in many fights”) or whether the adolescent is asked to rate his or her own behavior (“I get in many fights”) (see Supplementary Table 1 for the items included). Items of the ASR used to calculate antisocial personality problems in adults differ between versions over the years of data collection. For this study, we summed the 15 items that were available for all ASR questionnaires obtained in the NTR from 1997 until 2013 (see Supplementary Table for the items included). Petersen et al. [31] showed for externalizing problems measured by the CBCL, YSR, and ASR that there is theoretical and empirical support for construct validity invariance and, therefore, for examining these measurements over time.

### Statistical analyses

Mean age was calculated in SPSS (version 21). Average symptoms scores and their standard deviations were calculated using OpenMx [34]. The scores for childhood and adolescent conduct and adult antisocial personality problems were highly skewed, as is common for psychiatric symptoms in population-based samples. Therefore, to obtain accurate parameter estimations, the scores were divided into three roughly equal sized categories (low, middle, and high scores), and analyzed as categorical data with two thresholds [32]. In a threshold model, where the mean of the distribution is standardized at zero and the standard deviation one, it is assumed that the categorical trait has an underlying continuous distribution of liability [33] and polychoric correlations between twins reflect the correlations in liability. Polychoric twin correlations were estimated for MZm, DZm, MZf, DZf, and DOS pairs for childhood, adolescence, and adulthood using structural equation modeling in OpenMx [34]. With the full information maximum likelihood option, all available data were analyzed, including the data from the incomplete twin pairs. We estimated the 95% confidence interval around the correlations in OpenMx [35]. Sex differences in the prevalence of conduct and antisocial problems behavior at each age were investigated by testing whether thresholds could be constrained to be equal across sex. Next, we tested for quantitative sex effects on twin correlations by constraining the correlations of the male same-sex MZ and DZ twin pairs to be equal to the correlations of the female same-sex MZ and DZ twin pairs, respectively. If these constraints are not allowed, this indicates that the contribution of genetic and environmental influences may differ in males and females. Finally, to investigate whether different genes or different shared environmental factors operate in males and females, we tested whether the correlation for DZ same-sex and DOS twins could be constrained as a function of the DZ same-sex correlations.

Based on the outcomes of these analyses, we proceeded with the longitudinal analyses. First, the correlations between twin1 and twin2 across the three ages (i.e., cross-twin-cross-age correlations) were calculated. Next, in a genetic structural equation model, the observed phenotypic variance in each age group as well as the phenotypic covariance across age was partitioned into additive genetic (A), common environmental (C), and non-shared environmental (E) components [36]. MZ twins share (nearly) all their genetic material [37, 38], while DZ twins share, on average, 50% of their segregating genes. Therefore, a higher MZ than DZ twin correlation indicates that genetic factors play a role. When the DZ twin correlations are higher than half of the MZ twin correlation, there is resemblance among twins from the same family that is attributable to common environmental influences shared by children from the same family. Variation that is not due to genes or the common environment shared by twins is attributed to unique environment. In a similar vein, the genetic, shared environmental, and unique environmental influences to the stability of conduct and antisocial personality problems across the ages were estimated based on the cross-twin-cross-age correlations [39]. We derived the estimates for the heritability, the environmental effects, and the correlations between the genetic and environmental factors across the ages from the cross-twin-cross-age correlations after testing for the significance of A and C by the likelihood-ratio test by comparing an ACE model to an AE model for children, adolescents, and adults. In the likelihood-ratio test, the negative log-likelihood (−2LL) of the more constrained submodel is subtracted from the −2LL of the more general model. The difference between the two models follows a *χ* ^2^ distribution, where the number of *df* (degrees of freedom) is equal to the difference in *df* between the two models. Constraints were retained when they did not significantly deteriorate the fit (*p* < 0.01 due to multiple testing), so that the most parsimonious model is selected.

## Results

### Descriptives

Table 1 provides the mean ages, standard deviations, and age ranges of the twins included in the childhood and adolescent conduct problems groups and in the adult antisocial personality problems group. The untransformed mean symptom scores, standard deviations, and the two thresholds for the liability distributions for the three age groups are given for boys and girls separately. As expected, at all ages, males scored higher, which is reflected by significant differences in the thresholds (*p* < 0.001, model 2 in Table 3).

**Table 1:** Mean age, standard deviations (SD), and age range for children, adolescents, and adults

	Conduct problems								Antisocial personality problems			
	Children, CBCL				Adolescents, YSR				Adults, ASR			
Mean age (SD)	10.00 (0.44)				15.77 (1.31)				29.39 (11.12)			
Min–max	8.71–12.98				13.00–18.00				18.00–64.98			

	M	SD	Th1	Th2	M	SD	Th1	Th2	M	SD	Th1	Th2
Males	1.57	2.39	−0.10	0.42	3.10	2.58	−0.57	0.38	1.84	2.24	−0.45	0.62
Females	0.85	1.53	0.28	0.82	2.38	2.23	−0.22	0.73	1.51	1.90	−0.33	0.80

Childhood conduct problems were measured by the CBCL, adolescent conduct problems were measured by the YSR, and adult antisocial personality problems were measured by the ASR. The lower part shows the untransformed mean symptom scores, the standard deviations (SD), and thresholds (Th1 and Th2) based on an underlying normal distribution of liability estimated for the three age groups and separately for males and females

*CBCL* Child Behavior Checklist, *YSR* Youth Self-Report, *ASR* Adult Self-Report

### Twin correlations

Polychoric twin correlations and their 95% confidence intervals are shown in Table 2. For all three age groups, MZ correlations were higher than DZ correlations, suggesting that additive genetic factors play a role. The DZ correlations were larger than half the MZ correlations during childhood, suggesting shared environmental effects. During adolescence and adulthood, the DZ correlations were larger than half of the MZ correlations for males, but not for females. However, further testing showed that there were no significant differences (*p* > 0.01) between the correlations for same-sex male and female twin pairs (i.e., there were no quantitative sex differences), or between the DZ same-sex and DOS twins (i.e., there were no qualitative sex differences) as can be seen from Model 3 and 4 in Table 3. MZ and DZ correlations in the most parsimonious model are depicted in the middle of Table 2. The phenotypic correlation between childhood and adolescent conduct problems was 0.20, between adolescent conduct problems and adult antisocial personality problems 0.38, and between childhood conduct problems and adult antisocial personality problems 0.22. Table 2 also gives the cross-twin-cross-age correlations for the MZ twins (below the diagonal) and the DZ twins (above the diagonal). These cross-twin-cross-age correlations were higher for MZ twins than for DZ twins, indicating that genetic factors influence the stability of conduct and antisocial personality problems across the ages.

**Table 2:** Sample sizes and the polychoric twin correlations per age-by-zygosity-by-sex group, as well as correlation estimates constrained to be the same across sex (for MZ and DZ twin pairs)

	Conduct problems				Antisocial personality problems	
	Children		Adolescents		Adults	
	*N*	Twin correlation	*N*	Twin correlation	*N*	Twin correlation
MZm	1656	0.90 (0.87–0.91)	1012	0.47 (0.40–0.54)	1057	0.44 (0.35–0.51)
DZm	1587	0.68 (0.64–0.72)	928	0.32 (0.22–0.41)	763	0.34 (0.22–0.45)
MZf	1880	0.85 (0.83–0.88)	1429	0.52 (0.45–0.57)	2388	0.41 (0.35–0.46)
DZf	1466	0.66 (0.61–0.71)	1201	0.27 (0.18–0.35)	1481	0.22 (0.14–0.29)
DOS	3194	0.65 (0.61–0.68)	2269	0.21 (0.14–0.27)	2220	0.24 (0.14–0.34)
MZ		0.88 (0.86–0.89)		0.50 (0.45–0.54)		0.42 (0.37–0.46)
DZ		0.66 (0.63–0.69)		0.25 (0.20–0.29)		0.25 (0.19–0.31)
Children	9783	–		0.11 (0.09–0.11)		0.11 (0.09–0.11)
Adolescents	3977	0.18 (0.17–0.19)	6839	–		0.16 (0.13–0.16)
Adult	2665	0.22 (0.21–0.23)	2298	0.30 (0.30–0.31)	7909	–

The overlapping sample sizes across age and the cross-twin-cross-age correlations are depicted at the bottom of the table, where the MZ correlations are below the diagonal and the DZ correlations above the diagonal

**Table 3:** Model fitting statistics for the three age groups

			Estimated parameters	−2LL	*df*	Compared to	*X* ^2^	*p* value
Children	1	Saturated	9	34417.66	19,476	–	–	–
	2	Equal thresholds across sex	7	34896.08	19,478	1	478.43 (2)	<0.001
	3	MZm = Mzf & DZm = DZf	7	34427.56	19,478	1	9.91 (2)	0.01
	4	DZ = DOS	6	34429.02	19,479	3	1.46 (1)	0.23
Adolescents	1	Saturated	9	25285.53	11,937	–	–	–
	2	Equal thresholds across sex	7	25541.73	11,939	1	256.21 (2)	<0.001
	3	MZm = Mzf & DZm = DZf	7	25287.28	11,939	1	1.75 (2)	0.42
	4	DZ = DOS	6	25290.91	11,940	3	3.63 (1)	0.06
Adults	1	Saturated	9	23338.24	11,092	–	–	–
	2	Equal thresholds across sex	7	23378.17	11,094	1	39.93 (2)	<0.001
	3	MZm = Mzf & DZm = DZf	7	23341.73	11,094	1	3.49 (2)	0.18
	4	DZ = DOS	6	23341.75	11,095	3	0.02 (1)	0.89

For each model, the negative log-likelihood (−2LL) is given, with the number of degrees of freedom (df). The more restrained models are compared to models containing a larger number of parameters and tested with a Chi-squared test. The saturated (model 1) includes all correlations, as reported in Table 2. In model 3, the correlations between monozygotic males (MZm) and females (MZf) and the dizygotic males (DZm) and females (DZf) were constrained to be equal (quantitative sex differences). In model 4, the correlations between dizygotic same-sex (DZ) and opposite-sex (DOS) correlations were constrained to be equal (qualitative sex differences)

### Genetic and environmental influences

An AE model yielded a worse fit than the ACE model in childhood (*p* < 0.001), while in adolescence and adulthood, the AE model did not lead to a deterioration in fit compared to an ACE model (adolescents: *p* = 0.55, adults: *p* = 0.46). The estimates of the proportions of variance explained by genetic and environmental factors, their standard errors, and the genetic and unique environmental correlations across childhood and adolescent conduct problems and adult antisocial personality problems of the final longitudinal model are reported in Table 4.

**Table 4:** Standardized estimates of additive genetic (A) and common and unique environmental (C and E) influences and their 95% confidence intervals (CI)

	Model	A	C	E	Correlations		
					Children	Adolescents	Adults
Children	ACE	43% (38–44%)	44% (39–45%)	13% (12–14%)	–	0.07	0.03
Adolescents	AE	49% (45–51%)	–	51% (50–55%)	0.39	–	0.14
Adults	AE	43% (39–44%)	–	57% (53–61%)	0.49	0.67	–

Below the diagonal on the right the genetic correlations between the phenotypes assessed in children, adolescents and adults are given, and above the diagonal, the unique environmental correlations between the three different ages are presented for the most parsimonious longitudinal model

Genetic (43%) and shared environmental (44%) factors were equally important contributors to individual differences in conduct problems measured in 9–10-year-old twins. During adolescence, the effect of the shared environment disappeared and genetic influences explained 49% the variance. Roughly similar results were obtained in adulthood, with a heritability estimate of 43%. The effect of the unique environment increased from 13% in childhood to 51% in adolescence and 57% in adulthood. Genetic and non-shared environmental influences accounted for 91 and 9%, respectively, for the stability between childhood and adolescent conduct problems. Genetic and non-shared environmental influences accounting for 96 and 4% for the stability between childhood conduct problems and adult antisocial personality problems. Finally, genetic and non-shared environmental influences accounting for 80 and 20% for the stability between adolescent conduct problems and adult antisocial personality problems. These findings correspond with genetic correlations of 0.39 between childhood and adolescent conduct problems, of 0.67 between adolescent conduct problems and adult antisocial personality problems, and of 0.49 between childhood conduct problems and adult antisocial personality problems (Table 4). The non-shared environmental correlations ranged between 0.03 and 0.14. Thus, there is considerable genetic continuity, especially between adolescent conduct problems and adult antisocial personality problems.

## Discussion

Our aim was to explore the genetic architecture of conduct and later antisocial personality problems in childhood, adolescence problems, and adulthood in a unique longitudinal twin data set, collected over a period of over 27 years. At all ages, we observed the expected sex differences in mean symptom scores, with males scoring higher than females (differences in mean scores equaled about half the standard deviation for children and adolescents and a sixth of the standard deviation for adults). However, no quantitative or qualitative sex differences in genetic architectures were found, i.e., the proportions of variance explained by the genome did not differ between sexes and the same genes seemed to be expressed in males and females. Across ages, we found large differences in the influences of shared and unique environmental factors on variation in conduct and later antisocial personality problems. In 9–10-year-olds, genetic and *shared* environmental factors were equally important, explaining 43% and 44% of the individual differences in conduct problems. During adolescence and adulthood, the effect of the shared environment on individual differences in conduct and antisocial personality problems was non-significant and the genetic and *unique* environmental effects accounted for 49% and 51% in adolescents and 43% and 57% in adults. The phenotypic correlations across the ages varied between 0.20 and 0.38, showing childhood and adolescent conduct problems and adult antisocial personality problems are moderately stable. The genetic correlations were substantial across the ages, namely, 0.39 between childhood and adolescent conduct problems, 0.67 between adolescent conduct problems and adult antisocial personality problems, and 0.49 between childhood conduct problems and adult antisocial personality problems. The unique environmental correlations were far lower, ranging between 0.03 and 0.14.

In line with earlier studies, the heritability of childhood and adolescent conduct problems and adult antisocial personality problems is substantial (between 43% and 49%) [10–13, 40, 41], and genetic factors are the main contributor to covariation across the ages [14–17]. In agreement with an earlier study in 7-year-old Dutch twins, the influence of the shared environment on conduct problems in childhood was large (44%) [30]. Strikingly, the shared environmental effect was non-significant during adolescence and adulthood, while the sample sizes (6839 adolescent twin pairs and 7909 adult twin pairs, overlap of 3977 twin pairs) were sufficiently large to detect shared environmental influences [42, 43]. This is a different finding than reported by some earlier studies on conduct problems, which found small, at max 23%, but significant shared environmental influences on adolescent conduct problems and adulthood antisocial personality problems [10–17, 40, 44], and on the stability between the ages [15–17]. This difference might be due to differences in the assessment of the phenotype, or may reflect country-shared environment interactions. In Dutch twins, the decrease in the influence of C after childhood has also been reported for anxiety problem [45] and obsessive compulsive problems [46].

How should we interpret the disappearance of the shared environmental influences? It could mean that the shared environment is not of importance anymore after childhood, for example, because the span of control of the parents decreases. We speculate that the shared environmental factors that explain differences in conduct problems during childhood may include factors that have a protective effect that lose their influence during adolescence due to the changed parental role. This speculation is based on the finding that inadequate parental monitoring is a risk factor for the development of child and adolescent conduct problems [47]. Parental monitoring often decreases from childhood to adolescence as a natural development in the process of raising children [48–52]. A recent study in children-of-twins confirmed that parental knowledge of their children’s whereabouts, activities, and behaviors is a parental influence that diminishes adolescent externalizing behavior after accounting for genetic influences [53]. Continued parental monitoring and parental knowledge may not only be effective during childhood (when the shared environment plays such an important role), but as well during adolescence.

Future research on environmental factors should consider that environmental factors can be correlated with an individual’s genotype, i.e., gene–environment correlations. This describes the process, whereby an individual’s exposure to an environmental factor depends on the individual’s genotype. For example, a preference for peers with externalizing problems can be associated with a genetic predisposition for externalizing problems. For future research on environmental influences on conduct problems, children-of-twins and adoption studies are genetically informative designs that offer possibilities to account for such gene–environment correlations [54, 55].

Another factor, apart from age, that could explain the observed differences in environmental influences throughout development may be the change in rater. Typically, psychopathology in childhood is assessed by parents, whereas in adolescence and adulthood, self-ratings are feasible. During childhood, both twins within a twin pair were rated by their mother in our study, whereas in adolescence and adulthood, the twin and co-twin rated themselves; i.e., there were two raters per pair. This change in the number of raters can influence estimates of heritability and the shared environment, as Kan et al. [56] demonstrated. When both twins are rated by the same informant, any rater specific variance is added to the genetic and the shared environmental influences. However, when each twin is rated by a different informant, the rater specific variance is added to the unique environmental effect, resulting in a decrease in both the heritability and the shared environmental estimate. In our current study, we observed only a decrease in the amount of variance explained by shared environment and no decrease in heritability. Thus, our results suggest that the contribution of shared environmental influences may truly decreases with age. A change of rater also raises the question whether the parental and self-reports are measurement invariant, i.e., whether they measure the same underlying trait across age [57]. As shown in Supplementary Table 1, the items included in the CBCL and YSR to assess conduct problems are highly similar. However, highly similar items do not necessarily imply that the items have identical meaning for mothers and adolescents. To our knowledge, no study has addressed construct validity invariance for the conduct problem scales of the CBCL and YSR. However, Petersen et al. [31] argued on the basis of five conditions that there is theoretical and empirical support for construct validity invariance for the externalizing scales of the CBCL and YSR, a scale including all items used in the conduct problem scales (CBCL: 16 out of 33, YSR: 17 out of 30) that were used in the current study [31]. As (1) the measures were derived empirically, (2) showed a similar factor structure across time, (3) showed strong cross-time consistency, (4) strong convergent and discriminant validity over time with respect to internalizing problems, and (5) the items showed high internal consistency at each age, they concluded that examining the changes in externalizing problems as measured in the CBCL and YSR over time is permitted. Therefore, it seems unlikely that measurement non-invariance between mothers and adolescents fully explains the difference in the estimates for the contribution of C.

Besides the effect of age and the change of rater, the influence of the behavior of one twin on the behavior of the other twin may have become stronger during adolescence. If an increase in problems in one twin causes a decrease in the behavior of the other twin (contrast effect), this can result in an underestimation of the shared environment during adolescence. Twin contrast effects can be detected by analyzing whether there are prevalence differences between the MZ and DZ twins [58, 59]. We did not observe such differences as a function of zygosity: 44.43% of the 9–10-year-old MZ boys scored ‘low’ on conduct problems, compared to 43.64% of the DZ boys (for girls, MZ: 60.6% vs. DZ: 62.17%). For adolescent MZ boys, 29.81% scored ‘low’ vs. 26.76% of the DZ boys (for girls, MZ 44.43% vs. DZ 40.13%) and for adult males, 36.32% scores ‘low’ vs. 31.21% of the DZ males (for females, MZ 37.45% vs. DZ 36.32%). These were the largest differences that were observed and none were significant (*p* < 0.01 due to multiple testing). For ‘middle’ and ‘high’ scores, the differences in prevalence between MZ and DZ twins were even smaller. Thus, contrast effects between twins also do not appear to have caused an underestimation of the shared environmental influence in any of the age groups.

A limitation of the present study is that the ASEBA questionnaire symptom scores were skewed, as is common for psychiatric symptom scales. We, therefore, analyzed the data with a threshold model, which resulted in more accurate parameter estimates, but in lower statistical power [32]. This was balanced by the large samples, which also provided the opportunity to fully explore sex effects.

In conclusion, this study confirms a substantial genetic influence on conduct and antisocial personality problems across age and an important contribution of the shared environment on childhood conduct problems. There is a moderate stability in conduct problems and antisocial personality problems across the lifespan and genetic factors are the main contributor to this stability over the ages. These findings show the important role of genetic factors across the lifespan and of the shared environment during childhood on conduct problems.

## Electronic supplementary material

Below is the link to the electronic supplementary material.

Supplementary material 1 (PDF 50 kb)
