# Supplementary material for: Genetic and environmental influences on conduct and antisocial personality problems in childhood, adolescence, and adulthood
Source: Eur Child Adolesc Psychiatry. 2017 Jun 21;27(9):1123–32. doi: 10.1007/s00787-017-1014-y (PMC6133103; doi:10.1007/s00787-017-1014-y)
Supplement: Supplementary file 1 — Supplementary material 1 (PDF 50 kb) [file 787_2017_1014_MOESM1_ESM.pdf]

Supplementary material

Table 1. The list of items included in the sum score of conduct problems as measured in the Child Behavior Checklist (CBCL) and Youth Self-Report (YSR) and of antisocial personality problems as measured in the Adult Self-Report (ASR).

CBCL and YSR	ASR
Mean to others	I am mean to others
Damage or destroy things belonging to others	I damage or destroy things belonging to others
Lacks guilt	I get along badly with my family
Gets in fights	I get in many fights
Hang around people who get in trouble	I hang around people who get in trouble
Lies/cheats	I lie or cheat
Physically attacks people	I physically attack people
Leaves home	My behavior is irresponsible
Sets fire	I do things that may cause me trouble with the law
Stealing from home	I steal
Stealing outside home	I argue a lot
Swearing	I have a hot temper
Threatens to hurt people	I threaten to hurt people
Truant	I fail to pay my debts or meet other financial responsibilities
Breaks rules**	I break rules at work or elsewhere
Vandalism*	
Cruel to animals*	

*only CBCL ** only YSR
